# Efficacy of oxycodone in managing visceral pain among women undergoing repeat cesarean section: a randomized controlled trial

**DOI:** 10.3389/fmed.2026.1787893

**Published:** 2026-04-17

**Authors:** Qiwei Chen, Shishi Hu, Jie Liu, Guangyou Duan, Mengjie Chen, Shiqi Li

**Affiliations:** 1 Department of Anesthesiology, The Second Affiliated Hospital, Chongqing Medical University, Chongqing, China; 2 Department of Anesthesiology, Chongqing Hospital of Jiangsu Province Hospital, Chongqing, China; 3 Department of Gynecology and Obstetrics, The Second Affiliated Hospital, Chongqing Medical University, Chongqing, China

**Keywords:** cesarean section, neutrophil-to-lymphocyte ratio (NLR), numerical rating scale (NRS), oxycodone, sufentanil

## Abstract

**Objective:**

The global cesarean section (CS) rate is on the rise, with ratio of repeat CS becoming more common in China following the two-child policy. Postoperative visceral pain, particularly intense in repeat CS parturients and exacerbated by oxytocin, remains inadequately managed. Oxycodone, acting via κ-opioid receptors, has shown promise in relieving visceral pain, but its efficacy in repeat CS populations is unclear, which was explored in this study.

**Methods:**

This randomized controlled trial enrolled 100 parturients undergoing elective repeat CS, randomized to patient-controlled intravenous analgesia (PCIA) with oxycodone (50 mg/100 ml) or sufentanil (100 μg/100 ml). Primary outcomes were maximum visceral pain Numerical Rating Scale (NRS) scores during 0–24 h postoperatively. Secondary outcomes included visceral pain NRS during oxytocin administration on postoperative days 1–2, postoperative inflammatory markers (neutrophil-to-lymphocyte ratio, NLR), time to autonomic activity and PCIA presses. This trial was registered with the Chinese Clinical Trial Registry (ChiCTR2400087624; July 31 2024)

**Results:**

Significantly lower maximum visceral pain NRS scores in the oxycodone group during 24h after surgery compared to sufentanil group (2.2 ± 0.6 *vs*. 3.4 ± 0.7, *P* < 0.001), and also at all time points during oxytocin infusion (*P* < 0.001). The oxycodone group also had shorter time to autonomic activity (23.7 ± 5.1 vs. 26.3 ± 3.4 h, *P* = 0.004), lower postoperative NLR (7.2 ± 2.1 vs. 9.1 ± 3.7, *P* = 0.003), and fewer PCIA presses (*P* = 0.002). Multivariate linear regression confirmed oxycodone as an independent predictor of reduced maximum postoperative pain (adjusted β = −1.13, 95%CI:−1.41 to−0.85, *P* < 0.001).

**Conclusion:**

In conclusion, oxycodone effectively alleviates postoperative visceral pain and mitigates inflammatory responses in repeat CS parturients, making it a preferred choice for postoperative analgesia in this population.

## Introduction

Globally, the proportion of cesarean section (CS) in obstetric delivery has demonstrated a sustained upward trend (1). In China, hospital-based statistics report a cesarean rate of 44.1%. Concurrently, with the implementation of the two-child policy, the incidence of repeat CS is expected to increase proportionally, warranting focused research on its clinical implications (2). Cesarean section is frequently associated with significant postoperative pain (3), encompassing both incision pain and visceral pain, which is an inevitable consequence of the surgical procedure. Currently, compared to other aspects of postoperative pain management, the treatment of visceral pain following cesarean section has received relatively limited attention (4, 5). This type of pain is not only intense but has the potential to evoke negative emotional responses in parturients (6). Conversely, prior research has demonstrated that women undergoing a second cesarean section exhibit heightened pain sensitivity compared to those undergoing their initial cesarean section. Additionally, several studies have indicated that the intensity of visceral pain following a second cesarean section surpasses that experienced by primiparous women (7–9). Notably, the postoperative administration of oxytocin exerts a significant influence on the degree of visceral pain after cesarean section. Consequently, for the distinct cohort of women who have undergone a second cesarean section, there is a compelling need to investigate more targeted intervention strategies for managing postoperative visceral pain.

Currently, despite the implementation of numerous clinical interventions aimed at reducing postoperative pain following cesarean section, the prevalence of severe pain remains substantial (10). This persistent high incidence of severe pain significantly impedes the postoperative recovery process of women who have undergone cesarean section (11, 12). Currently, opioid medications, including sufentanil, remain among the most frequently employed intravenous agents for postoperative analgesia following cesarean section. But their effect on visceral pain is not significant. Oxycodone, a semi-synthetic opioid medication, is derived from thebaine, an alkaloid compound. Animal studies have demonstrated that oxycodone exerts an inhibitory effect on visceral pain via κ-opioid receptors. Meanwhile, its analgesic effect is also closely associated with the activation of μ-opioid receptors (13). Clinical research findings indicate that oxycodone potentially exhibits greater advantages compared to other analgesic medications in relieving postoperative visceral pain, and demonstrates higher efficacy than μ-opioid receptor agonists, such as sufentanil (14–16). Consequently, it is hypothesized that oxycodone presents greater advantages in mitigating visceral pain subsequent to the second cesarean section.

Building on the aforementioned information, a randomized controlled trial (RCT) was carried out to examine the impact of oxycodone on the alleviation of uterine contraction—induced pain in women undergoing a subsequent cesarean section. The present study is projected to contribute to the further refinement of postoperative analgesia regimens for parturients who have undergone cesarean section. This endeavor is geared toward enhancing the comfort levels of parturients during the postoperative phase and expediting their recovery process, thereby facilitating their return to optimal physical and functional states.

## Materials and methods

### Research design

This study was designed following the CONSORT statement and employed a 1:1 parallel-group, randomized, controlled, double-blind research design. This study was conducted in compliance with the Declaration of Helsinki. It was reviewed and approved by the Institutional Ethics Committee of Chongqing Hospital of Jiangsu Province Hospital on July 25, 2024 (Approval No.: 2024 Kelun Review 011). All participants provided written informed consent prior to enrollment. This trial was registered with the Chinese Clinical Trial Registry (ChiCTR2400087624; July 31 2024) prior to patient enrollment. The full protocol and all participant-identifiable data are available from the corresponding author upon reasonable request.

### Patients

All patients underwent screening in the obstetric ward prior to surgery and were recruited 1 day before surgery in accordance with the inclusion and exclusion criteria. The inclusion criteria for this study were as follows: 1) Patients scheduled to undergo elective repeat cesarean section under spinal anesthesia; 2) Willingness to participate in the study and provision of written informed consent. The exclusion criteria were as follows: 1) Patients with coagulation dysfunction; 2) Patients with an American Society of Anesthesiologists (ASA) physical status classification of grade III or above; 3) Patients with concurrent central nervous system diseases; 4) Patients with chronic painful conditions; 5) Patients with long-term use of anti-inflammatory and analgesic drugs; 6) Patients with allergies to the drugs used in this experimental study.

### Anesthesia and analgesia techniques

Upon admission to the operating room, patients were monitored for electrocardiogram (ECG), blood pressure (BP), and pulse oxygen saturation (SpO_2_). Prior to surgery, 3 ml of peripheral venous blood samples were collected for routine blood tests, and inflammatory markers were recorded, specifically including white blood cell count, neutrophil count, and lymphocyte count. Patients were placed in the lateral decubitus position. Standardized combined spinal-epidural anesthesia (CSEA) was administered at the L3-L4 intervertebral space. A total volume of 3.0 ml, consisting of 2.0 ml of 0.75% ropivacaine mixed with cerebrospinal fluid, was injected, followed by placement of an epidural catheter at a depth of 3–5 cm. All surgeries were performed by a team of surgeons with extensive clinical experience using standardized surgical techniques. This surgical team has successfully completed more than 1,000 cesarean sections to date. Postoperatively, bilateral transversus abdominis plane block (TAPB) was performed as follows, each side received 15 ml of 0.25% ropivacaine as the local anesthetic. The intraoperative blood loss and operation time were recorded.

Immediately after the operation, patient-controlled intravenous analgesia (PCIA) was initiated via a patient-controlled infusion pump. According to sealed random number assignment, PCIA was administered to patients in each group using oxycodone or sufentanil (medication was administered immediately postoperatively for more than 24 h and had no significant impact on the neutrophil–to–lymphocyte ratio (NLR).), respectively. In the oxycodone group, 50 mg of oxycodone was mixed with 0.9% normal saline to a total volume of 100 ml and then loaded into the PCIA pump. In the sufentanil group, 100 μg of sufentanil was mixed with 0.9% normal saline to a total volume of 100 ml and loaded into the PCIA pump in the same manner. The parameters of the 100-ml PCIA pump were set as follows: continuous infusion rate of 3 ml/h, bolus dose of 3 ml, lockout time of 30 min, and maximum hourly dose of 12 ml. The numerical rating scale (NRS) scores for uterine contraction pain, resting wound pain, and dynamic wound pain, along with the number of PCA pump presses and PCA pump consumption (ml), were recorded at 0–6 h, 6–12 h, and 12–24 h postoperatively. The intensity of postoperative pain was monitored and recorded by the researchers. For the prevention of postoperative nausea and vomiting (PONV), 8 mg of ondansetron was added to the analgesic pump.

### Randomization and allocation hiding

In this study, simple randomization was employed, with random numbers generated by an independent assistant not involved in data collection or analysis using an online random number generator. The assistant prepared the allocation sequence and concealed the numbers in opaque, numbered, and sealed envelopes. Patients undergoing cesarean section were randomized into the oxycodone or sufentanil group according to the random numbers in the envelopes, which were opened in the operating room. Postoperative analgesia strategies were then implemented accordingly. Subsequently, the envelopes were resealed and stored at the study site until completion of the research.

Different analgesic regimens were administered by specialized pain physicians using patient-controlled infusion pumps of the same model. Throughout the study, all patients and researchers, including the surgical team, were blinded to the group allocation and analgesic regimens. Neither the independent assistant nor the pain specialists were involved in data collection and remained independent of the final data analysis. Preoperative and intraoperative data collection, as well as follow-up during postoperative hospitalization, were conducted by trained investigators who were not involved in group assignment.

### Outcomes measurement

The Numerical Rating Scale (NRS) was used to assess and record the following parameters: the intensity of uterine contraction pain, resting wound pain, and dynamic wound pain, along with the number of PCA pump presses and PCA pump consumption (ml) at 0–6 h, 6–12 h, and 12–24 h postoperatively. The NRS was also applied to evaluate the pain intensity on the first and second postoperative days, as well as at 5 min, 10 min, 20 min, 30 min, 40 min, 50 min, and 60 min after intravenous oxytocin administration.

In this study, the primary outcomes were defined as the intensity of uterine contraction pain at 0–6 h, 6–12 h, and 12–24 h postoperatively, as well as at 5 min, 10 min, 20 min, 30 min, 40 min, 50 min, and 60 min after intravenous oxytocin administration on the first and second postoperative days. Secondary outcome measures included: 1) the degree of postoperative incision pain; 2) the time to onset of postoperative breakthrough pain; and 3) preoperative and postoperative hematological inflammatory markers, specifically white blood cell count, neutrophil count, and lymphocyte count.

### Statistical analysis

This trial was a randomized controlled trial, with the primary outcome being the intensity of postoperative uterine contraction pain. Based on previous research data, the mean numerical rating scale (NRS) score for uterine contraction pain in parturients undergoing repeated cesarean section receiving conventional analgesics (sufentanil) was 3.9 ± 1.3. It was estimated that oxycodone could reduce uterine contraction pain by 20%, with a 1:1 parallel control design applied. Using PASS sample size calculation software, with a significance level of 0.05 and a power of 0.80, the required sample size was calculated to be 43 cases per group. Considering a 15% loss to follow-up rate, 50 cases were planned to be enrolled in each group, resulting in a total of 100 subjects required.

Statistical analysis was performed using SPSS 22.0 software. General descriptive statistics were applied based on data types: continuous variables with a normal distribution were expressed as mean ± standard deviation; non-normally distributed quantitative data were presented as median (interquartile range); and qualitative variables were expressed as counts (percentages). For intergroup comparisons, the independent samples *t*-test, Mann-Whitney U test, or chi-square test was used according to the data types, respectively. Single-factor and multi-factor linear regression analysis were sequentially performed to determine the role of group intervention (oxycodone *vs*. sufentanil) on uterine contraction pain NRS. In single-factor linear regression analysis the variable with *P* < 0.1 were included in the multi-factor linear regression analysis. Regression coefficient with 95% confidence interval (CI) was also calculated. A *P*-value < 0.05 was considered statistically significant.

## Result

**Figure 1:**
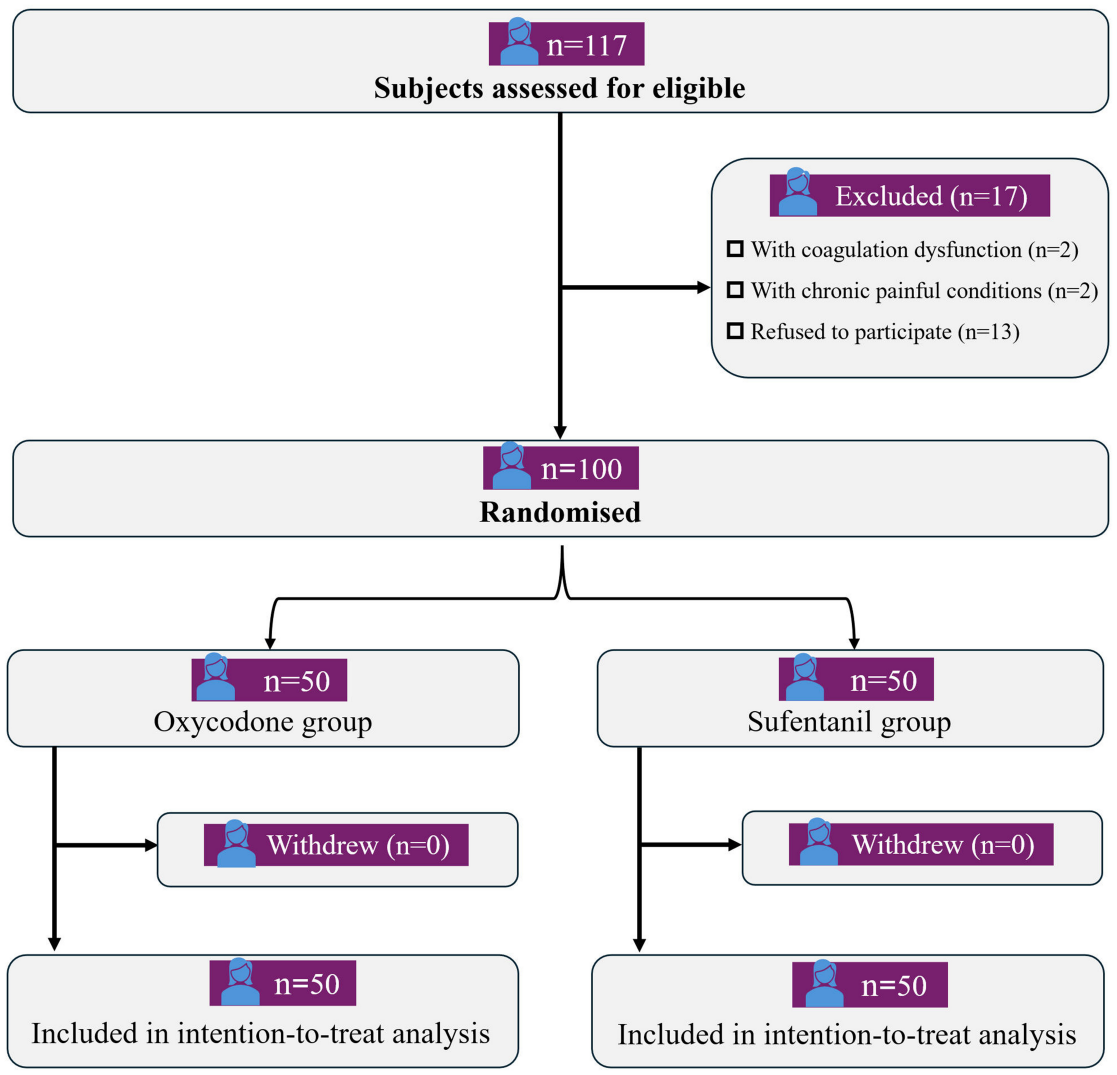
Research Flow Diagram.

As shown in Figure 1, a total of 100 cesarean women completed the study and were included in the final analysis. The baseline demographic, preoperative and intraoperative data were listed in Table 1, time from last gestational period in Oxycodone group was significantly longer than that in Sufentanil group (9.2 ± 3.8 *vs*. 7.5 ± 3.7year, *P* = 0.023). And no other significant difference was found between cesarean women in Oxycodone group and Sufentanil group.

**Table 1:** Demographic, preoperative and intraoperative data.

Characteristics	Oxycodone group (*n* = 50)	Sufentanil group (*n* = 50)	*P* value
Age (year)	32.8 ± 4.1	31.6 ± 3.9	0.141
Height (cm)	159.7 ± 4.6	160 ± 3.6	0.682
Weight (kg)	76.7 ± 7.1	75.8 ± 5.7	0.501
BMI (kg/m^2^)	30.1 ± 2.6	29.6 ± 2.4	0.358
Gestational period (week)	38.7 ± 0.8	38.6 ± 0.8	0.603
Time from last Gestational period (year)	9.2 ± 3.8	7.5 ± 3.7	0.023
WBC	8.2 ± 2.1	8.3 ± 1.6	0.835
NEUT	6.0 ± 1.7	6.1 ± 1.4	0.681
NLR	4.1 ± 1.0	4.4 ± 1.4	0.162
Duration of the operatio n(min)	48.2 ± 16.4	49.5 ± 16.5	0.685
Blood loss (ml)	368 ± 74	389 ± 136	0.342
Level of anesthesia≥T6	28(56%)	35(70%)	0.147
Height of newborn (cm)	50 ± 1.6	50.3 ± 1	0.233
Weight of newborn (g)	3370.6 ± 440.5	3383.4 ± 399.6	0.879

Data were presented as Mean ± SD or numbers (percentage); BMI, body mass index; WBC, white blood cell; NEUT, neutrophil count; NLR, neutrophil-to-lymphocyte ratio.

**Figure 2:**
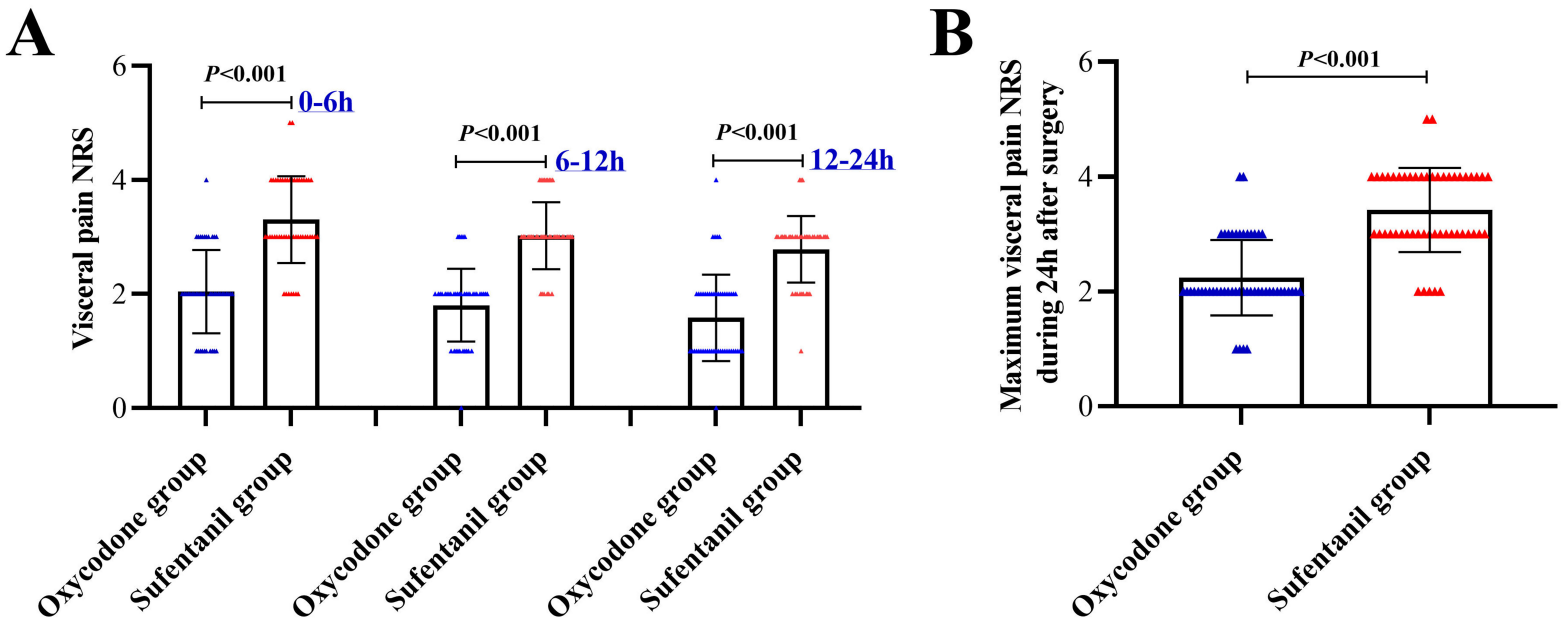
Comparisons of Visceral Pain Severity Between Two Groups. **(A)** Comparisons of Visceral Pain at Different Time Points; **(B)** Comparison of Maximum Visceral Pain at 24 Hours after surgery; NRS, number rating scale.

Comparisons of visceral pain NRS at different time points were presented in Figure 2A, all of them at 0–6h [2.0(2.0–2.3) *vs*. 3.0(3.0–4.0), *P* < 0.001], 6–12h [2.0(1.0–2.0) *vs*. 3.0(3.0–3.0), *P* < 0.001] and 12–24h [1.0(1.0–2.0) *vs*. 3.0(2.0–3.0), *P* < 0.001] in Oxycodone group was significantly lower than those in Sufentanil group. And the primary outcome, i.e., maximum visceral pain NRS during the 24h after surgery [2.0(2.0–3.0) *vs*. 3.0(3.0–4.0), *P* < 0.001] in Oxycodone group was significantly lower than those in Sufentanil group (Figure 2B).

**Figure 3:**
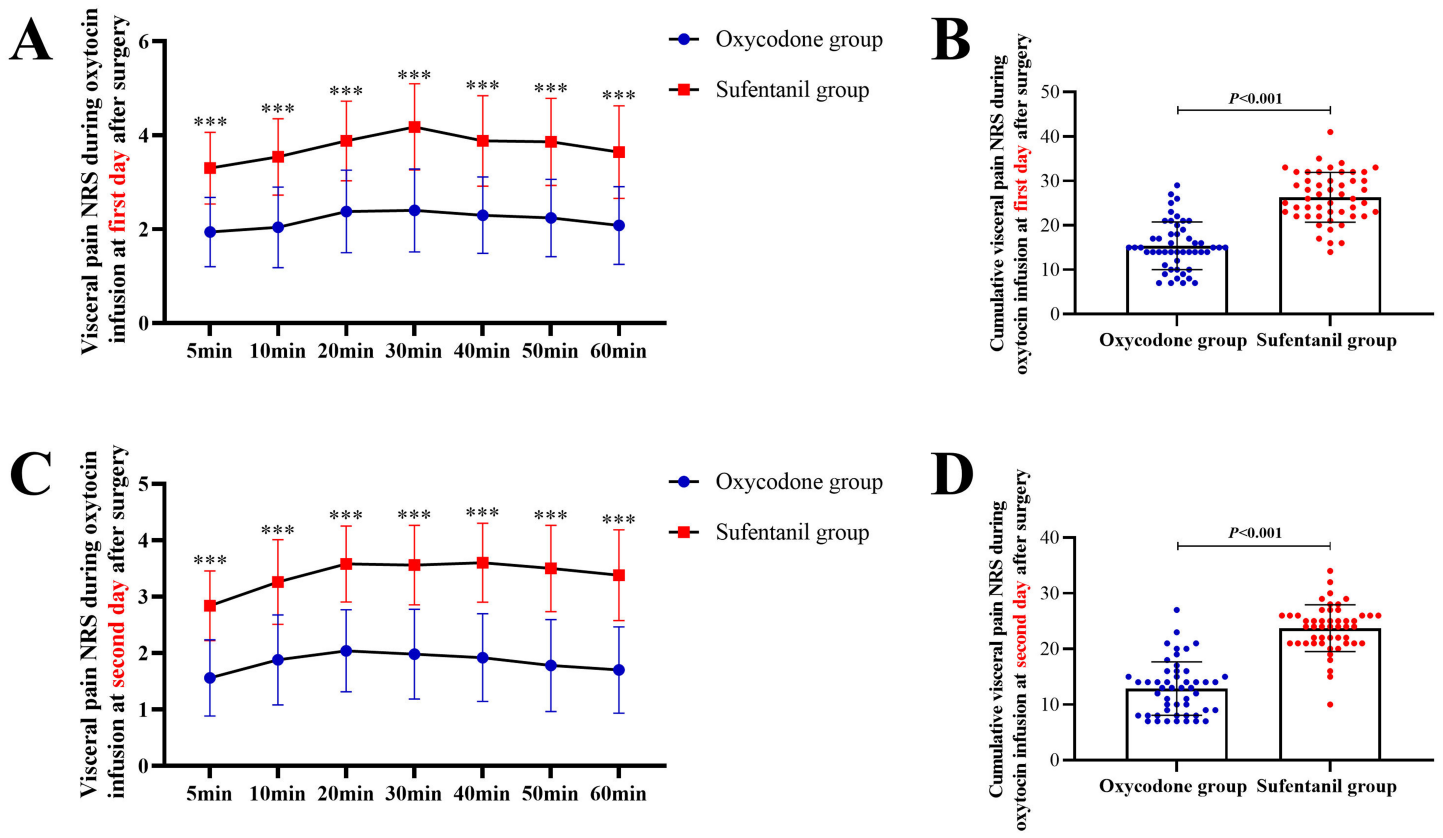
Comparison of Visceral Pain Severity Between Two Groups During Oxytocin Infusion. **(A)** Comparison of visceral pain at different time points during infusion on postoperative day 1; **(B)** Comparison of cumulative visceral pain during infusion on postoperative day 1; **(C)** Comparison of visceral pain at different time points during infusion on postoperative day 2; **(D)** Comparison of cumulative visceral pain during infusion on postoperative day 2; NRS, number rating scale.

In addition, as shown in Figures 3A, C visceral pain NRS during oxytocin infusion at both of first and second day in Oxycodone group were significantly lower than those in Sufentanil group. The cumulative visceral pain NRS at first day [15.0(11.8–18.3) *vs*. 26.0(22.0–30.5), *P* < 0.001] and second day [13.0(8.0–15.3) *vs*. 24.0(21.0–26.0), *P* < 0.001] after surgery were all significantly lower in Oxycodone group compared to Sufentanil group (Figures 3B and 3D).

As listed in Table 2, the time to autonomic activity (23.7 ± 5.1 *vs*. 26.3 ± 3.4 h, *P* = 0.004) in Oxycodone group were significantly lower than those in Sufentanil group. Both of the NLR at 24h after surgery (7.2 ± 2.1 *vs*. 9.1 ± 3.7, *P* = 0.003) and ratio of NLR at 24h after surgery compared to that before surgery (1.80 ± 0.53 *vs*. 2.09 ± 0.59, *P* = 0.016) in Oxycodone group were significantly lower than those in Sufentanil group. And PCIA pressing frequency in Oxycodone group was 0.0(0.0–0.0), which was significantly less than that in Sufentanil group 0.0(0.0–1.0) (*P* = 0.002). No other significant differences were found between two groups.

**Table 2:** Postoperative data between two groups.

Characteristics	Oxycodone group (*n* = 50)	Sufentanil group (*n* = 50)	*P* value
Time to feel visceral pain (h)	0.5 (0.5–0.6)	0.5 (0.5-1.0)	0.420
Time to autonomic activity (h)	23.7 ± 5.1	26.3 ± 3.4	0.004
WBC at 24h after surgery	11.7 ± 2.7	12.1 ± 2.6	0.505
NEUT at 24h after surgery	9.4 ± 2.2	9.9 ± 2.4	0.240
NLR at 24h after surgery	7.2 ± 2.1	9.1 ± 3.7	0.003
Ratio of WBC at 24h after surgery compared to that before surgery	1.46 ± 0.26	1.49 ± 0.35	0.609
Ratio of NEUT at 24h after surgery compared to that before surgery	1.64 ± 0.36	1.69 ± 0.44	0.516
Ratio of NLR at 24h after surgery compared to that before surgery	1.80 ± 0.53	2.09 ± 0.59	0.016
PCIA pressing frequency	0.0 (0.0–0.0)	0.0 (0.0−1.0)	0.002
PCIA consumption (ml)	65.0 ± 4.5	66.3 ± 4.3	0.157
Apgar score at 1min	9.9 ± 0.1	9.9 ± 0.4	0.365
Apgar score at 5min	10.0 ± 0.0	9.9 ± 0.2	0.159
Apgar score at 10min	10.0 ± 0.0	9.9 ± 0.1	0.322
Postoperative nausea and vomiting	6 (12.0%)	11 (22.0%)	0.183
Blood loss at 12h after surgery(ml)	316 (123–492)	275 (105–473)	0.149
Hospital stay (day)	5.5 ± 0.7	5.6 ± 1.2	0.467

Data were presented as Mean ± SD, medium (interquartile range) or numbers (percentage); WBC, white blood cell; NEUT, neutrophil count; NLR, neutrophil-to-lymphocyte ratio.

Effects of grouped factors on maximum pain NRS during 24 h after surgery determined by logistic analysis was shown in Table 3. The results showed that Age (*P* = 0.027), gestational period (*P* = 0.099), height of newborn (*P* = 0.077) and group factor (oxycodone *vs*. sufentanil) (*P* < 0.001) were included in the multi-factor linear regression analysis. In the multi-factor linear regression analysis only group factor (oxycodone *vs*. sufentanil) showed significant effect on maximum pain NRS during 24 h after surgery, and the adjusted regression coefficient was−1.13(95%CI:−1.41 to -0.85).

**Table 3:** Effects of patient characteristics and grouped factors on maximum pain NRS during 24 h after surgery.

Variables	Regression coefficient (95%CI)	*P* value	Adjusted regression coefficient (95%CI)	*P* value
Age (year)	−0.05 (-0.09 to−0.01)	0.027	−0.02 (-0.06 to 0.02)	0.328
BMI (kg/cm^2^)	0.03 (-0.04 to 0.11)	0.854		
Gestational period (week)	−0.19 (-0.43 to 0.04)	0.099	−0.15 (-0.34 to 0.04)	0.120
Time from last Gestational period (year)	−0.04 (-0.09 to 0.01)	0.084	0.01 (-0.04 to 0.05)	0.831
Duration of the operation (min)	0.00 (-0.01 to 0.01)	0.804		
Blood loss (ml)	0.00(0.00 to 0.00)	0.236		
Height of newborn (cm)	0.12 (-0.01 to 0.26)	0.077	0.07 (-0.04 to 0.18)	0.188
Weight of newborn (g)	0.00(0.00 to 0.00)	0.215		
Group (oxycodone *vs*. sufentanil)	−1.18 (-1.46 to−0.90)	<0.001	−1.13 (-1.41 to−0.85)	<0.001

BMI, body mass index; CI, confidence interval.

In the present study, no severe opioid-related adverse events, including respiratory depression, were observed in either parturients postoperatively or newborns following delivery. In addition, this study performed statistical analyses of newborn Apgar scores at birth and parturient blood loss at 12 h postoperatively, and no statistically significant differences were observed.

## Discussion

This study demonstrated that the visceral pain Numerical Rating Scale (NRS) score of parturients in the experimental group was lower than that in the control group at all observed time points. In addition, during the period of oxytocin administration on postoperative day 1 (POD1) and postoperative day 2 (POD2), the visceral pain NRS scores of parturients in the experimental group were also lower than those in the control group. Furthermore, the neutrophil-to-lymphocyte ratio (NLR) of the experimental group was lower than that of the control group after surgery.

Visceral pain is among the main sources of pain during cesarean section. As established by previous studies, parturients undergoing repeat cesarean section often suffer from more intense visceral pain than primiparas undergoing primary cesarean section (8, 17, 18). Unlike most previous investigations, this study thus specifically included the population of parturients undergoing repeat cesarean section. Lin et al. conducted a randomized controlled trial (RCT) focusing on postoperative analgesia in patients undergoing laparoscopic radical resection of colorectal cancer, and their results indicated that oxycodone exerted a more potent effect than sufentanil in relieving visceral pain (19). Another meta-analysis further confirmed that oxycodone exhibits superior efficacy in relieving postoperative visceral pain compared with other opioid medications (16). This could be related to the mechanism by which oxycodone activates κ-opioid receptors—receptors that play a role in the generation of visceral pain (20, 21). However, it remains unclear whether oxycodone still retains such advantages in parturients who may experience more severe visceral pain following repeat cesarean section. Our experimental results showed that, during the entire postoperative observation period (including the 0–6 h, 6–12 h, and 12–24 h time intervals), the visceral pain Numerical Rating Scale (NRS) scores of the experimental group were significantly lower than those of the control group. This result suggests that oxycodone is a better choice than sufentanil for alleviating visceral pain in individuals (specifically parturients) who have undergone repeat cesarean section.

The number of PCIA compressions was significantly different between groups in this study. Although statistically significant, this finding should be interpreted with caution in clinical practice. A reduced PCA compression count reflects more steady postoperative analgesia, suggesting the analgesic protocol better satisfies patients' personalized pain requirements and may alleviate perioperative stress while enhancing comfort. However, with a mere 1 ml difference in total consumption between groups, the clinical impact of this statistical difference is likely modest.

In addition, given that oxytocin administration during the postoperative management of clinical parturients is a major factor contributing to the exacerbation of visceral pain, this study, unlike previous clinical trials, conducted real-time and detailed assessments of visceral pain severity during the postoperative oxytocin application period. Such a design not only allows for a more accurate comprehension of patients visceral pain conditions during special time periods but also enables the comparison of clinical effect discrepancies between oxycodone and sufentanil in the alleviation of visceral pain. Observational findings from this study indicated that, during the 1-h observation period after oxytocin infusion, dynamic evaluations conducted at seven time points showed that, for parturients undergoing repeat cesarean section, the visceral pain NRS scores in the experimental group were lower than those in the control group at every time point, and the proportion of severe visceral pain cases in the control group exceeded that in the experimental group. This finding further verifies that oxycodone produces a highly significant effect in alleviating postoperative visceral pain among the special group of women with a history of repeat cesarean section.

Cesarean section-induced tissue trauma is capable of triggering acute systemic inflammation, and this inflammatory response exerts a significant effect on the occurrence of acute pain (22). The neutrophil-to-lymphocyte ratio (NLR) not only acts as a reliable biomarker for reflecting the body's immune response to various infectious and non-infectious stimuli, but also has been widely applied in multiple medical fields associated with infection, inflammation, and stress, owing to its simple detection method. Additionally, previous studies have confirmed that NLR can serve as an objective indicator for predicting pain severity (23–27). In the context of a systemic or local inflammatory state, neutrophils function as the primary line of defense. They are capable of fighting infections via two key mechanisms: phagocytosis and the release of neutrophil extracellular traps (NETs) (28, 29). Acute stress exposure is associated with a decline in the number of lymphocytes within the body (30). The neutrophil-to-lymphocyte ratio (NLR), as a comprehensive marker encompassing two key inflammatory components, can effectively reflect the equilibrium between neutrophils and lymphocytes in the context of complex inflammatory responses. Simultaneously, variations in the NLR ratio are able to predict and influence the occurrence of postoperative acute pain in patients (27, 31). Therefore, in the present study, changes in the neutrophil-to-lymphocyte ratio (NLR) can be used to reflect differences in the immune status of parturients before and after surgery, and this indicator is also associated with the occurrence of postoperative acute pain in parturients. Previous clinical studies have confirmed that, in comparison with sufentanil, oxycodone is capable of regulating the levels of inflammatory cytokines and mitigating postoperative inflammatory responses (32). However, more direct inflammatory indicators remain required in future study. In addition, there was no statistically significant difference in the incidence of adverse events between the two groups, demonstrating that oxycodone exhibited a safety profile similar to that of sufentanil. Drawing on the results of this study and the above pieces of evidence, it can be objectively concluded that oxycodone exhibits advantage compared with sufentanil in postoperative analgesia for parturients undergoing repeat cesarean section.

It should be noted that this study still has certain limitations. First, the selection of research subjects is limited: this experiment only enrolled parturients undergoing repeat cesarean section as research participants, and the analgesic effect, dose safety, and mechanism of action of the intervention (oxycodone) for visceral pain in primiparas after cesarean section have not yet been clarified, which restricts the application scope of the research conclusions. Secondly, this study focused on the analysis of short-term indicators, including pain scores and inflammatory factor levels, but did not conduct follow-up observations on rehabilitation-related indicators—such as the incidence of long-term pain and postoperative complications in parturients, as well as the long-term health outcomes of mothers and infants. Therefore, the comprehensive impact of oxycodone on the postoperative rehabilitation of parturients undergoing repeat cesarean section remains to be further investigated. Thirdly, this study adopted a single-center research design, which lacks cross-validation with multi-center data. In the future, multi-center, large-sample prospective studies should be conducted to improve the external validity of the conclusions and enhance their clinical reference value.

In conclusion, our results demonstrate that oxycodone is effective in relieving postoperative visceral pain and reducing the inflammatory response following repeated cesarean section. Accordingly, oxycodone may represent a valuable alternative for postoperative analgesic management in these patients.

## Data Availability

The raw data supporting the conclusions of this article will be made available by the authors, without undue reservation.
